# Successful treatment of MSSA acute bacterial prostatitis using dalbavancin

**DOI:** 10.1093/jacamr/dlae003

**Published:** 2024-01-22

**Authors:** Athena L V Hobbs, Michael S Gelfand, Dennis Marjoncu

**Affiliations:** Department of Pharmacy, Cardinal Health Innovative Delivery Solutions, 7000 Cardinal Place, Dublin, OH 43017, USA; University of Tennessee Health Science Center, College of Medicine, Memphis, 910 Madison Ave, Ste 1031, Memphis, TN 38163, USA; Department of Pharmacy, Methodist Le Bonheur Healthcare, 1265 Union Ave, Memphis, TN 38104, USA

To the Editor-in-chief:

Acute bacterial prostatitis (ABP) is characterized by acute inflammation of the prostate gland that can present as genitourinary pain, voiding symptoms and sexual dysfunction. Gram-negative microorganisms such as *Escherichia coli* are the predominant pathogens; however, the incidence of ABP caused by Gram-positive pathogens such as *Staphylococcus* spp. and *Streptococcus* spp. has been increasing recently.^1^ ABP complications can include prostatic abscess, chronic bacterial prostatitis, metastatic infection, epididymitis and bacteraemia.^2^ Empirical treatment of ABP should ensure adequate prostate penetration and Enterobacterales coverage before being de-escalated pursuant to urine culture and susceptibility results.^3^ Recommendations for treatment of ABP caused by Gram-positive pathogens are sparse; thus, clinicians are left to determine the best agents based on spectrum of activity, drug penetration and activity in the pH range at the site of infection. Additionally, social determinants can complicate treatment regimens, though dalbavancin has been used successfully for earlier discharge of vulnerable patients.^4^ We present a case of a patient with MSSA prostatitis successfully treated with dalbavancin.

## Case

**Figure 1.**
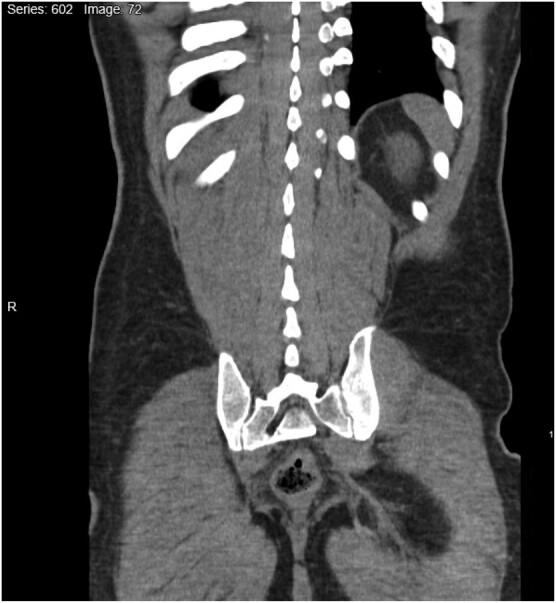
CT scan showing prostatitis with probable prostatic abscess.

A middle-aged male person who injects drugs (PWID) presented to the emergency department (ED) following 3 days of lower back pain, urinary urgency, frequency, dysuria and constipation. He also reported a 2 day history of chills, vomiting and decreased oral intake. Notably, he had no specific risk factors for ABP. In the ED, the urinalysis was negative for nitrites and leucocyte esterase, but positive for WBCs and trace bacteria. A CT scan of the abdomen showed prostatitis with probable prostatic abscess (Figure 1). On examination, his prostate was non-tender and had no areas with fluctuance. The urology team recommended empirical initiation of levofloxacin 750 mg once daily for 7 days. When admission blood cultures yielded MSSA, levofloxacin was de-escalated to cefazolin 2 g every 8 h on Day 4 and continued through to Day 9, when the patient was discharged (Table 1). Infectious diseases (ID) was consulted and ordered a trans-thoracic echocardiogram (TTE), which showed no signs of endocarditis, and an MRI of the spine, which showed no signs of vertebral osteomyelitis. ID was unable to determine whether ABP was the source or a consequence of the bacteraemia, but no other potential sources were identified and urology determined that an attempted drainage was not indicated. ID elected to treat this patient for a minimum of 4 weeks for *Staphylococcus aureus* ABP and complicated bacteraemia with probable prostate abscess.

**Table 1.** Blood culture susceptibility results

	*S. aureus*	
Antimicrobial agent	Interpretation	MIC (mg/L)
Cefazolin	Susceptible	≤4
Ceftaroline	Susceptible	≤0.5
Clindamycin	Susceptible	≤0.25
Daptomycin	Susceptible	≤0.5
Erythromycin	Susceptible	≤0.25
Linezolid	Susceptible	≤2
Oxacillin	Susceptible	≤0.25
Trimethoprim/sulfamethoxazole	Susceptible	≤0.5/9.5
Vancomycin	Susceptible	≤1

In light of dalbavancin’s excellent coverage of MSSA, as well as the ability to use vancomycin as a surrogate for *Staphylococcus* spp., ID started the patient on dalbavancin, with the first 1500 mg dose given immediately prior to discharge.^5,6^ The ID team did not feel comfortable recommending oral agents due to the complexity of this case, the patient’s social history, and lack of oral antibiotics that could be tolerated through the completion of therapy. A second dose was administered 14 days later in the outpatient infusion centre for a total of 4 weeks of dalbavancin therapy. The urinalysis on the day of the second dalbavancin infusion remained concerning, with a small amount of blood in the urine, as well as leucocyte esterase, bacteria and WBCs; however, the patient did not express any complaints suggesting persistent or unresolved infection at the 3 week clinic follow-up, and repeat imaging showed improvement of the abscess.

## Discussion

Walker *et al.*^7^ reported the first use of dalbavancin in prostatitis in 1 of 21 patients receiving dalbavancin off-label. The patient received 8 weeks of therapy, and the infection subsequently resolved. De Pablo-Miro and colleagues^8^ also described clinical outcomes in patients treated with dalbavancin, seven of which had a urinary tract infection (UTI), prostatitis or an abdominal infection. Though this study does not isolate prostatitis individually when describing results, it is apparent that at least one patient in this study was treated with dalbavancin for prostatitis. To the authors’ knowledge, this is the first case report specifically detailing the course and outcomes of a patient with MSSA ABP and bacteraemia successfully treated with dalbavancin. The ID team opted to use dalbavancin 1500 mg every 14 days for two doses in light of prolonged tissue concentrations above the MIC_90_ for *S. aureus* and clinical efficacy in deep-seated Gram-positive infections in previous trials.^9,10^ Previous studies illustrate that dalbavancin may have a role in treating complicated Gram-positive bacteraemia in PWID, and there is currently a multicentre, randomized open-label study enrolling patients with *S. aureus* bacteraemia including infective endocarditis to complete therapy with dalbavancin versus standard of care after blood cultures clear.^11,12^

Dalbavancin’s favourable pharmacokinetic profile positions it to be a consideration for ABP caused by Gram-positive pathogens. Initial review shows urine clearance of dalbavancin is 33.5% in healthy volunteers.^13^ Its high volume of distribution (*V*_d_) of 1.06–1.6 L/kg translates to excellent tissue penetration.^14^ There are scant data regarding dalbavancin prostate penetration; thus, clinicians must extrapolate from other agents. Dalbavancin has the same volume of distribution as levofloxacin, which has excellent distribution into prostate tissue.^14,15^ Knowing that *V*_d_ is only one aspect, we also compared dalbavancin and levofloxacin’s ability to penetrate different sites and found they both penetrate skin, bone, blister fluid and synovial fluid well.^14,15^ Notably, both the synovial fluid and the prostate are alkaline, with the pH of synovial fluid approximately 7.8 and the pH of prostatic fluid increasing from 7.31 in healthy men to 8.34 in the setting of infection, suggesting the drug is also active in the basic environment of the prostate.^16–18^ Indeed, though there is no literature to illustrate the impact of pH on dalbavancin’s activity, another lipoglycopeptide, oritavancin, is unaffected by pH when treating susceptible organisms but might have reduced activity against vancomycin-resistant *Enterococcus faecium* isolates in an acidic environment.^19^ Dalbavancin’s prolonged half-life, broad Gram-positive spectrum of activity, and safety profile make it an attractive option for outpatient treatment of a variety of infections, especially when oral or other parenteral agents are not ideal.^14,16^

In this case, our patient required hospitalization and IV antibiotic therapy for the treatment of ABP with probable prostatic abscess, which resulted in systemic symptoms. Upon resolution of bacteraemia, the patient’s only indication for continued hospitalization was the need for IV antibiotics as the ID team did not feel comfortable recommending oral agents, as mentioned previously. Use of dalbavancin allowed the patient to be discharged 4 weeks earlier than would have been possible without this option, which led to significantly lower hospitalization costs and greater patient satisfaction.
